# Smart Memory Storage Solution and Elderly Oriented Smart Equipment Design under Deep Learning

**DOI:** 10.1155/2022/6448302

**Published:** 2022-05-09

**Authors:** Chen Gong, Runze Liu, Nan Zhou, Jidong Luo, Deepak Kumar Jain

**Affiliations:** ^1^Department of Computer Science, School of Engineering, The University of Manchester, Greater Manchester M139PL, UK; ^2^College of Mechanical and Electronic Engineering, Tarim University, Tarim 843300, China; ^3^College of Automation, Chongqing University of Posts and Telecommunications, Chongqing, China

## Abstract

This study explores the memory characteristics of elderly individuals to design effective smart devices based on smart memory storage solutions under deep learning to improve the learning efficiency of elderly individuals. The different memory formation stages in the existing human brain are analysed. A smart memory storage solution based on memory-enhanced embedded learning is constructed based on meta-learning under deep learning, which reduces the cost of learning new tasks to the greatest extent. Finally, the performance of the proposed solution is verified using different datasets. The results reveal that the solution based on deep learning has obvious effects on different datasets, with an average accuracy rate of 99.7%. By synthesizing a large number of target sample features, this solution can lower the learning difficulty and improve the learning effect. The proposed elderly oriented smart device effectively reduces the shortcomings in the current market and lowers the learning difficulty, which provides an important reference for further enriching devices in the ageing market.

## 1. Introduction

Social ageing has gradually become prominent with the rapid development of Chinese society. According to statistics from relevant departments, the population over 65 years old in China will account for 14% of the total population by approximately 2022, realizing the transition to an ageing society [1]. Among the population with chronic diseases, elderly individuals account for a larger proportion. Accelerated ageing and the increasing number of people infected with chronic diseases have greatly hindered social development [2]. Especially for the elderly, the demand for chronic disease management is increasing. Chronic disease generally has a short onset period and a more rapid onset, which also seriously endangers the health of the elderly [3]. The development of traditional medical treatment is restricted by region and technology. However, smart medical methods are emerging with the development of the Internet of Things (IoT), deep learning, and global positioning system (GPS) technology. Compared with traditional medical methods, the core of smart medical care is healthy management and testing of the elderly, which can provide more convenient and efficient services for the elderly [4]. With the help of IoT technology and corresponding human sensors, smart medical care can realize the collection and analysis of patient-related body data. Smart medical care can effectively shorten the connection among patients, doctors, and hospitals and strengthen the mutual relationship of the three ultimately achieving timely prevention and timely treatment [5]. Although home smart health management equipment has been greatly improved in technology, there are still some problems in practical applications, which bring great difficulties to the adaptation of the elderly [6]. Therefore, designing corresponding elderly oriented smart devices according to the physical function of the elderly shows very important practical value for protecting the health of the elderly.

The new medical service model developed by smart medical care helps win-win cooperation for medical institutions, Internet companies, and smart medical product design companies, which provide patients with home smart health management products with better user experience [7]. The research and development of home medical equipment for the elderly in China is still in its infancy stage, and there are many operation difficulties encountered by the elderly in interacting with products in their daily lives. Some scholars have deeply analysed the advantages and disadvantages of current smart devices and proposed that the design has to ensure ease of use based on the user characteristics of the elderly. In addition, the intermediate operation process has to be reduced to make it more convenient, which helps to effectively monitor the health of the elderly [8]. Smart medical and health equipment can effectively prevent the deterioration of diseases to a certain extent and protect the physical safety of the elderly [9]. Deep learning is a branch of machine learning. It is an algorithm that uses an artificial neural network (ANN) as the architecture to perform characterization learning on data. It is an algorithm in machine learning based on characterization learning of data [10]. This method can update the device data according to the way the human body learns, which can effectively simulate the learning process of the elderly to a certain extent and is more convenient to implement. Therefore, some scholars have applied it to elderly oriented smart devices and obtained good experimental effects [11]. Against the background of ageing, domestic ageing is intensifying, and the speed of chronic diseases is accelerating among the elderly [12]. From the perspective of the industry background, the current more common smart health management terminal products are designed for young people, ignoring the memory and learning of the elderly, and reducing the utilization rate of products among elderly users [13]. Therefore, based on studying the memory and learning of the elderly, it is necessary to adopt a deep learning method to establish a design system for the ease of use of the smart health management terminal based on the memory and learning rules of the elderly.

In this study, deep learning methods are introduced from the perspective of memory storage to establish and design corresponding elderly oriented smart device solutions based on learning efficiency and memory research theories. In addition, a smart memory storage solution based on memory-enhanced embedded learning is proposed, the effectiveness of which is verified further through the performance analysis of the specific dataset. The basis of this study is to improve the learning efficiency of elderly users when using smart health management terminals, which can provide reliable ideas for the research and development of related smart devices. The first part of this study is an introduction to the background of smart medical development and the theory of memory research. The second part describes the current development of learning rate and memory theory through the introduction of recent related research work. The third part refers to the experimental method, which introduces the intelligent memory solution under deep learning in detail, and performs the intelligent device design and system architecture for the elderly. The fourth part shows the classification results of different datasets and the comparison results of different extraction networks. Finally, the fifth part draws the experimental conclusions, the comparative analysis results of different image datasets, and video datasets to verify the effectiveness of the solution.

## 2. Recent Related Works

### 2.1. Learning Rate

Learning efficiency is the amount of knowledge that can effectively be accepted and absorbed. Research on learning efficiency is mostly concentrated in the field of teaching. The foundation is to study the memory characteristics of the human body and adopt different strategies to effectively improve people's learning efficiency [14]. Fattinger et al. introduced a new deep sleep method used to locally disturb the motor cortex and found that when slow waves are selectively disturbed in the motor cortex, this recovery process can be greatly weakened, showing that deep sleep is a necessary condition to maintain sustainable learning efficiency [15]. Katona and Kovari developed a brain-computer interface (BCI) system and applied it to the learning efficiency test of cognitive neuroscience to evaluate output results by observing the vigilance level calculated by ThinkGear ASIC module technology and found that there is an obvious difference between this method and the conventional cognitive neuroscience test [16]. Pu et al. believed that the internal motivation of learners is the most critical determinant that affects the nature of the results, and critical thinking is essential for improving learning efficiency, which was proven through specific experiments [17]. Ma et al. used a clustering strategy to divide the population into multiple clusters and proposed an orthogonal learning framework to improve its learning mechanism; the experiment proved that this method is very effective in optimizing complex functions and improving learning efficiency [18]. These studies analysed the relationship between different types of memory materials in educational disciplines on the working memory and learning of different subject types, confirmed the important position of memory in high-level cognitive activities, and reflected that memory has a great influence on learning efficiency. The psychological and physiological effects of the elderly on some specific factors are correspondingly reduced, so this study focuses on how to improve the learning efficiency of the elderly using smart health management terminal products to reduce their learning costs from the perspective of memory.

### 2.2. Memory Theory

Memory theory is a cognitive psychology theory that studies the occurrence, development, and laws of human memory. The earliest method was proposed by the psychologist Ebbinghaus for various memorization materials, which needed to adopt different methods of checking the storage capacity to improve the memory ability of the human body [19]. Subsequently, American scientists further refined the above theory and proposed that primary memory involves direct conscious experience, and its reappearance does not require effort but is a true reappearance of things that have just been noticed, so it is inseparable from the current consciousness and has a temporary nature [20]. Later, related scholars proposed a corresponding forgetting curve, which proposed that the secondary memory is a retelling of primary memory, in which only part of the primary memory can form the secondary memory, and the rest is forgotten by the human brain [21]. For the study of memory theory, Weger et al. used a systematic method to develop a taxonomy of mental processes involving recall and found that this method can provide support for human memory and that mental activities involving various types of recall are greatly different [22]. Sweller et al. (2019) introduced emotional memory from memory psychology into the field of product interaction design and established a basic emotional memory system architecture, proving the value of emotional memory in design innovation [23]. Kvavilashvili and Rummel noted that when people naturally engage in forward thinking without clear instructions, they considered upcoming tasks and planned activities rather than simulating specious but novel hypothetical scenes [24]. Although memory characteristics belong to the field of psychology, which is difficult to carry out quantitative research, the current research basically focuses on how to make the learning plans reasonably and effectively based on human memory characteristics, and scholars who combine memory theory and design theory for research are relatively rare.

## 3. Methods

### 3.1. Memory Process of Human

**Figure 1:**
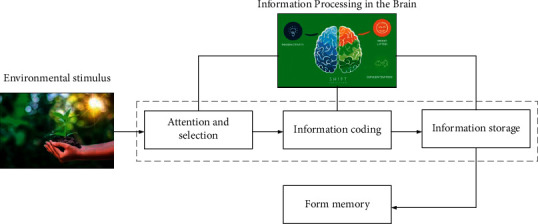
The main memory process in the human brain.

Learning and memory can be considered a process of combination, and the ability of the human brain to remember information will affect the learning efficiency of the human body to a certain extent. As shown in Figure 1, the main memory process in the human brain includes (1) attention and selection (the core of which is to form a certain cognitive ability through the perception of the surrounding environment), (2) information coding (the core of which is to realize effect combination and management of the obtained information), and (3) information storage (the core of which is to permanently record the sorted information and knowledge). Attention and selection are the first processes of memory. At any moment, there is much information in everyone's environment. In many cases, the human sensory system cannot process all the information, so it has to make a selection, which can be conscious or unconscious [25].

**Figure 2:**
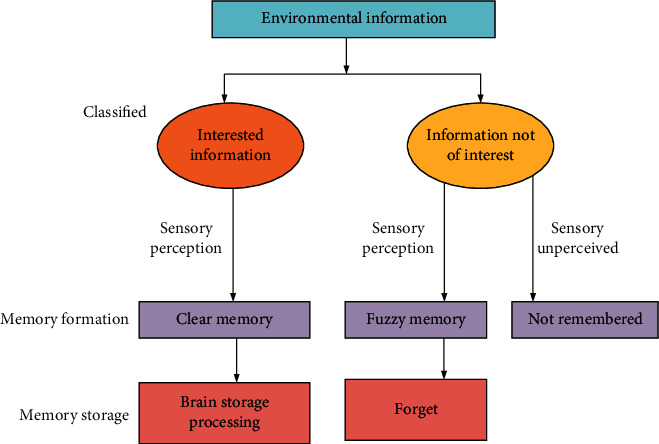
Information processing in the human brain.

If there is a large amount of information, the things that the human brain is interested in are perceived by the sensory system and stored as clear sensory memories, which are processed into long-term memories and stored. Things that the human brain is interested in are not perceived by the sensory system, forming vague sensory memory, and finally are forgotten. The specific process can be obtained in Figure 2. The information that the human brain consciously selects to remember is the information that people deliberately want to remember. Therefore, the key information that users need to perceive is required when a product is designed. Increasing the amount of information stimulus to the human senses can help the body to better perceive and remember this information [26].

Coding processes the information that has attracted the attention of the human brain. It is a complex and changeable process in the brain. Encoded information varies from person to person. For different types of information received by the sensory organs, the human brain has a different encoding process. For a single piece of information, humans use multiple sensory organs to receive information, so they remember the information more deeply, and the information content of the memory will be more accurate. The main reason for the decline in memory of the elderly with age is that the process of encoding information is weakened, especially those involving complex logical relationships that are difficult for the elderly to remember. Storage is storing the information in memory. The stored information forms short-term memory and long-term memory according to the length of time. Short-term memory refers to brief and short-term memory. Simple repetition or practice can help to convert short-term memory into long-term memory to a certain extent, and another strategy for conversion is deep thinking [27].

### 3.2. Smart and Memory Solutions under Deep Learning

**Figure 3:**
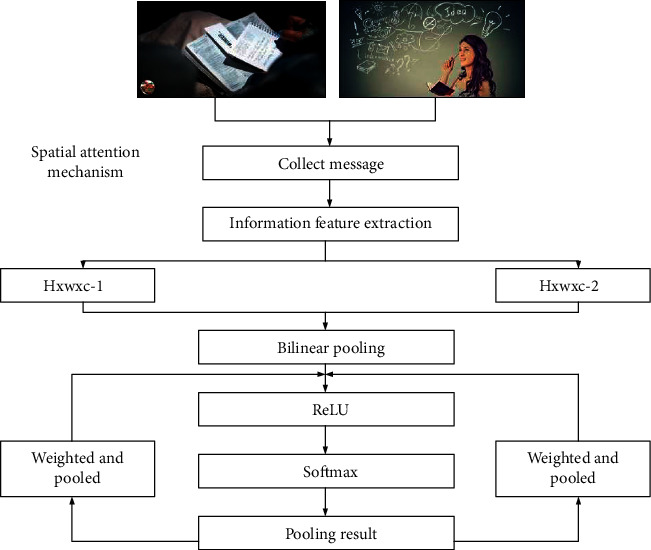
The mechanism of task-related spatial attention.

Based on the abovementioned memory process and the memory characteristics of the elderly, it is found that the deep learning method is more suitable for the memory analysis of the elderly, so the smart memory and storage solution of deep learning are introduced. As shown in Figure 3, the spatial attention mechanism is adopted to help the elderly distinguish different environments and establish corresponding feature aggregation images in space; then, two activation functions (ReLU and softmax) are used to convolve to eliminate irrelevant features and maintain the feature outcomes with the strongest relevance and output a corresponding soft attention weight result. At positions with smaller weights, the spatial attention enhancement feature representation method [28] is obtained by calculating the initialized characterization weight value.

The specific calculation method is given as Theorem 1.

**Theorem 1 .**

The calculation of the initial characterization weight is

(1) $$\begin{array}{c} S_{p}=\sum_{m,n}\alpha_{m,n}e_{m,n,p}. \end{array}$$

In the above equation, *S*_*P*_ refers to any element in the feature vector *S*, *a*_*m,n*_ represents any element and information, and *e*_*m,n*_,*p* represents any element in any initial character *E*.

Different from those methods that rely entirely on memory blocks to generate feature representations, memory blocks are undertaken as an auxiliary part in this study. For the categories in the meta-training set, a memory module *M* is trained, and the memory block is adopted in the meta-testing stage to enhance task-related representations. In the meta-training stage, the feature representations of the support set samples are continuously used to update the memory slots of their corresponding categories. A mechanism is designed in this study to avoid forgetting the previous memory when the memory is updated.

Specifically, the sample features of a support set enhanced by spatial attention are updated according to the following equation:

(2) $$\begin{array}{c} m_{\text{cls}}\leftarrow \frac{m_{\text{cls}}+\gamma^{\text{tcls}}S_{s}^{\left(i,j\right)}}{{\left\|m_{\text{cls}}+\gamma^{\text{tcls}}S_{s}^{\left(i,j\right)}\right\|}_{2}},t_{\text{cls}}=1. \end{array}$$

**Theorem 2 .**

In (2), *γ*^tcls^ is the attenuation rate, cls represents the category in the meta-training set, *m*_cls_ represents the representation of the category in the memory module, *t*_cls_ refers to the number of times the memory of the category is updated, and *S*_*s*_^(*i*, *j*)^ represents the support set sample feature enhanced by spatial attention. As memories about categories accumulate, the importance of newly added representations will decrease. The quantity of data in the meta-training set is fixed, and the probability of encountering repeated data becomes greater as the number of training rounds increases. In the early training stage, the memory relies to a large extent on newly added representations to update. With the accumulation of memory, the memory in the memory slot is more robust than the newly added representation, so the weight of the new representation needs to decrease with increasing time. The weight vector *w*_cls_ can be obtained by calculating the cosine similarity of each

(3) $$\begin{array}{c} w_{\text{cls}}=\text{Soft}\max\left(\frac{v\cdot m_{\text{cls}}}{\left\|v\right\|\cdot \left\|m_{\text{cls}}\right\|}\right). \end{array}$$

**Theorem 3 .**

In the equation above, *v* represents the function of obtaining the auxiliary vector of memory enhancement. The memory update is performed only in the meta-training stage. In the meta-test, only the read value of the memory is performed to support the dataset. The average value of the sample features of the supporting dataset can be calculated with equation (4) below:

(4) $$\begin{array}{c} C_{\text{cls}}^{\text{mean}}=\frac{1}{k}\sum_{i}S_{S}^{\left(i,j\right)}I\left(y_{S}^{i}=\text{cls}\right). \end{array}$$

In equation (4) above, *I*(*y*_*S*_^*i*^=cls) is the designated symbol, the value is 1 or 0, and *k* is the correlation coefficient. Next, the category representation can be calculated using the auxiliary vector

(5) $$\begin{array}{c} C_{\text{cls}}=\sigma \left(\alpha \right)C_{\text{cls}}^{\text{mean}}+\left(1-\sigma \left(\alpha \right)\xi \left(C_{\text{cls}}^{\text{mean}}\right)\right). \end{array}$$

In the above equation, *σ* is a sigmoid function, which is used to weigh the weight between the representation of spatial attention enhancement and the memory auxiliary vector. The characterization of the query sample set *cls* of the *K* vector can be calculated as follows:

(6) $$\begin{array}{c} Q_{j,\text{cls}}=\frac{1}{k}\sum_{i}S_{q}^{\left(j,i\right)}I\left(y_{s}^{i}=\text{cls}\right). \end{array}$$

**Figure 4:**
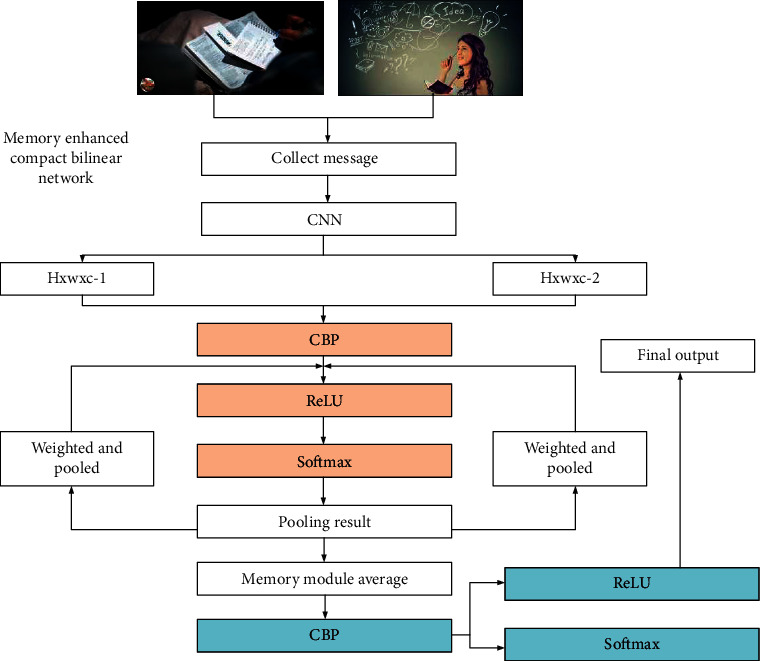
Memory-enhanced compact bilinear network.

Finally, the related tasks can be embedded, and the outputs can be regarded as two tuples. These two tuples will be input to the metric learning calibration block to learn the similarity metric among the respective elements. In the metric learning part, compact bilinear pooling is adopted so that all elements in the category feature can interact to obtain a comprehensive and compact fusion feature representation [29]. The specific structure is shown in Figure 4. Learning data are collected through deep learning neural networks, convolution, and pooling are performed through ReLU and softmax functions, and the feedback value of the final data result is output.

### 3.3. Elderly Oriented Smart Device

**Figure 5:**
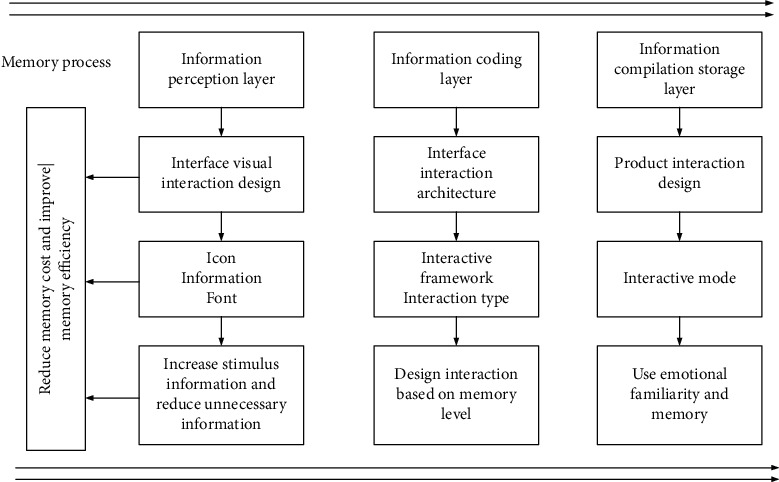
The specific details of the hierarchical model with an easy-to-use design.

Based on the abovementioned core memory process, the corresponding elderly oriented smart device is designed, which is divided into an information perception layer, an information coding layer, and an information storage layer. The core is the easy-to-use design of the home smart health management terminal for elderly users, which makes the product easy to learn and use for elderly users, with less memory burden and high user satisfaction. Improving product usability can be achieved by reducing product functions, streamlining interface information, or reducing user cognitive costs. Reducing the memory burden in the process of using the product is also the embodiment of the ease of use product design. Therefore, a hierarchical model is constructed in this study for the three stages of the information memory process in the human brain and the connection with the smart health management terminal products. The specific details of the model are shown in Figure 5. The level of information sensitivity helps the brain select information to a certain extent, and this information forms the basis for its attention to the environment and task execution. It is processed and assembled in stages. The information storage stage stores a permanent record after assembly and classification. First, the human brain is sensitive to a large quantity of data in the environment. Then, the data are stored in the deeper dimension of the information encoding. Finally, the data to be stored are encoded in the human brain. The process of storing information one by one in the brain gradually increases from shallow to deep.

### 3.4. Parameters Setting and Data Sources of the Model

The proposed model is verified on different datasets, including image datasets and video datasets. The image datasets include Omniglot (the first image dataset for small-sample classification), miniImageNet (containing 60,000 colour images in 100 categories) [30], and tieredImageNet (containing 779,165 images in 34 higher-level categories, which are subdivided into 20 training categories, 6 verification categories, and 8 test categories). The video datasets include UCF11 (a very challenging action recognition dataset, containing 1,600 videos in 11 action categories), UCF101 (an action recognition dataset, covering 101 different types of actions and a total of 13,320 videos), and HMDB51 (covering indistinguishable fine-grained categories such as smile, laughter, diet, and drinking) [31].

Task-based training is adopted in this study, and the meta-testing process is simulated by sampling the N-way K-shot task of the meta-training set [32]. In the Omniglot dataset, the size of the image is 28 × 28, and the image in the miniImageNet and tieredImageNet datasets is scaled to a size of 84 × 84. For all experiments in this section, the Adam optimizer is adopted, the initial learning rate is set to 0.001, and the learning rate is halved after 20,000 episodes. The training is stopped when there is no lift on the validation set. The memory calibration block is updated and extracted during the meta-training process, and the updated decay rate *y* is set to 0.995. In the meta-test, the update process of the memory calibration block is frozen, and only auxiliary vectors are extracted to enhance the representation of the category level. The bilinear pooling rate is set to 0.7%∼3%.

## 4. Results and Discussion

### 4.1. Classification of Image Data

**Figure 6:**
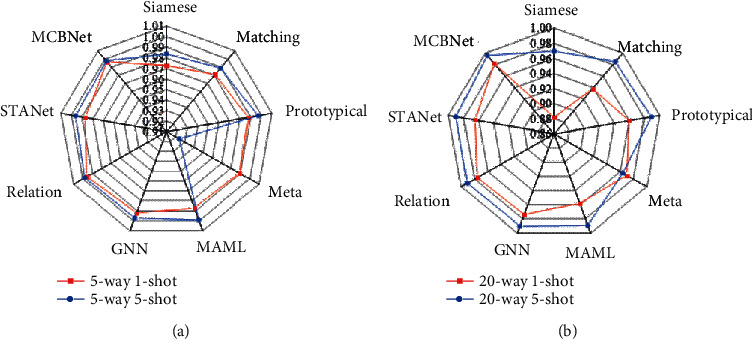
The sample classification results on the Omniglot dataset.

Figure 6 shows the sample classification results on the Omniglot dataset. Figure 6(a) illustrates the 5-way classification results, and Figure 6(b) shows the 20-way classification results. Except for the earlier Siamese network, other models achieve an accuracy rate greater than 90% on all tasks. On the simpler 5-way 1-shot and 5-way 5-shot tasks, many models, including the proposed solution, reach an accuracy rate of 99%. When the supporting samples of categories increase, the classification effect of all models improves, and the accuracy of all methods is lower than that of other tasks when the number of categories in the task increases from 5 to 20. In the most difficult 20-way 1-shot task, the solution proposed can achieve an accuracy rate of approximately 98%, which is approximately 0.6% higher than the best results in other methods.

**Figure 7:**
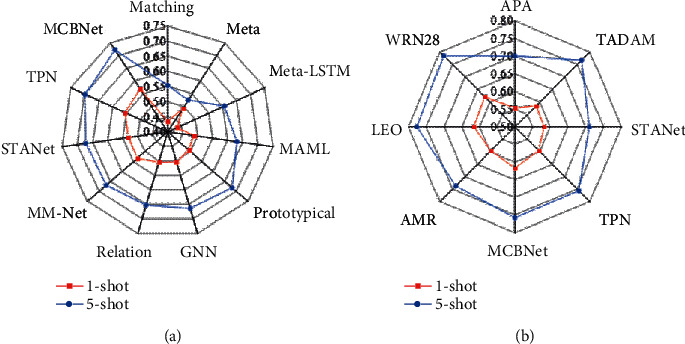
The sample classification results on the miniImageNet dataset.

Figure 7 illustrates the sample classification result on the miniImageNet dataset. Figure 7(a) shows the classification result on G64F-4, and Figure 7(b)shows the classification result on ResNet-12. Three types of feature extraction networks are used. When C64F-4 is adopted, the accuracy rate of the TPN solution is increased by approximately 1.4% and 2.4% on the 5-way 1-shot and 5-way 5-shot, respectively. When ResNet-12 is adopted, the accuracy rate on the 5-way 1-shot task is improved by approximately 2.1% and that on the 5-way 5-shot task is approximately 0.8% lower than that of TADAM. However, WRN-28 is adopted, and the accuracy rate of LEO improves by 0.4% and 1% on 5-way 1-shot and 5-way 5-shot tasks, respectively, in contrast to the previous models with good performance.

**Figure 8:**
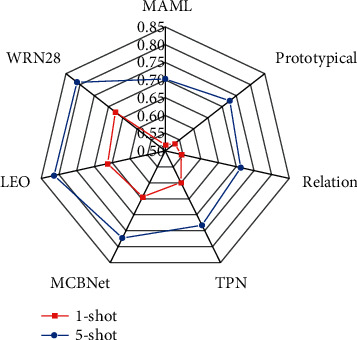
The sample classification results on the tieredImageNet dataset.

The sample classification results on the tieredImageNet dataset are illustrated in Figure 8 below. As the feature extraction network deepens, the gap between the 1-shot experimental group and the 5-shot control group decreases. In the meta-feature extraction network, the data accuracy of the experimental group is 49.2%, and the data accuracy rate of the control group is 52.6%. It shows that providing a linearly separable feature extraction method can perform deeper feature extraction on the feature extraction network, which demonstrates the advantages of the solution proposed in this study.

### 4.2. Classification of Video Datasets and Comparison of Different Extraction Networks

**Figure 9:**
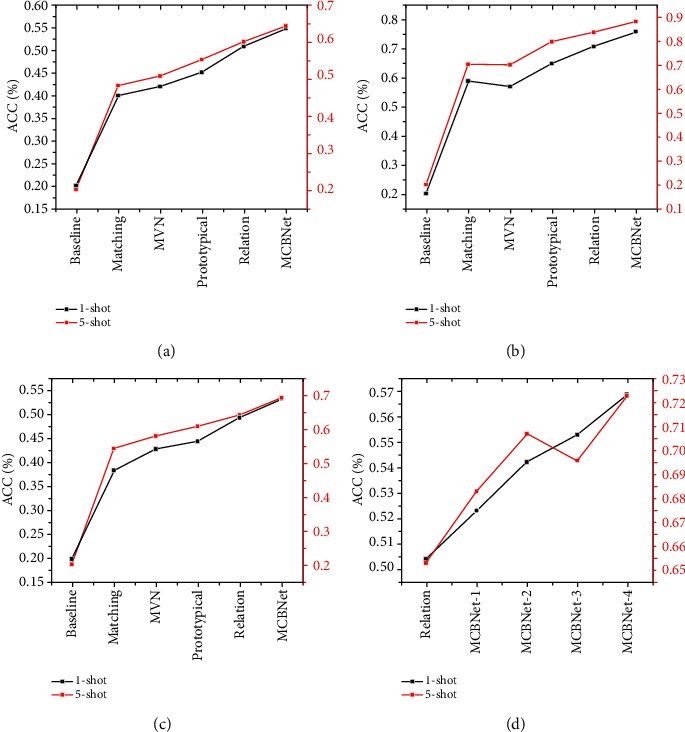
Classification results on different video datasets.

As shown in Figure 9, Figures 9(a)∼9(c) show the classification results on the UCF11, UCF101, and HMDB51 datasets, respectively. The number of categories in the dataset had a more obvious impact on the results. There were only 11 categories of the UCF11 meta-training set and meta-test, and its category division ratio was 6 : 5, which means that 5 categories were sampled in 6 categories during the training, so there were 6 possible combinations for the category space of the task. Therefore, compared to UCF101 with 101 categories, each model performs poorly on UCF11. Second, the differences among the dataset categories also have some impacts on the results. In contrast to UCF101, the categories of HMDB51 are more fine-grained, and the quality of videos is uneven, so the performance of each calibration type on HMDB51 is relatively poor. The multimodal compact bilinear net (MCBNet) solution using deep learning still has a large improvement over the relational network. Figure 9(d) shows the results of ablation experiments of different experimental solutions. The accuracy rate of the proposed solution improves by approximately 1.8% on the 1-shot task and nearly 3% on the 5-shot task.

**Figure 10:**
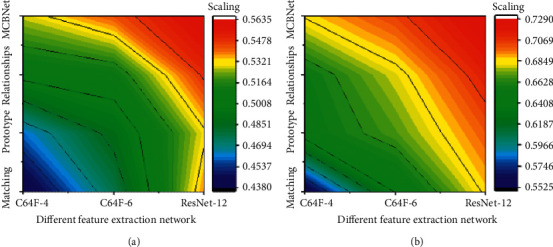
Comparison of different extraction networks on the datasets.

Figure 10 illustrates the comparison results of different extraction networks. Figures 10(a) and 10(b) show the results under 1-shot and 5-shot conditions, respectively. Under different deep neural networks (DNNs), the advantage of the proposed solution is that the feature extraction network shrinks gradually during deepening. This is essentially because the linear separability of the feature increases with the deepening of the feature extraction network, so even a fixed distance metric can show the similarity well in this case.

### 4.3. Discussion on the Results of Comparison between Learning Ability and Program Performance

**Figure 11:**
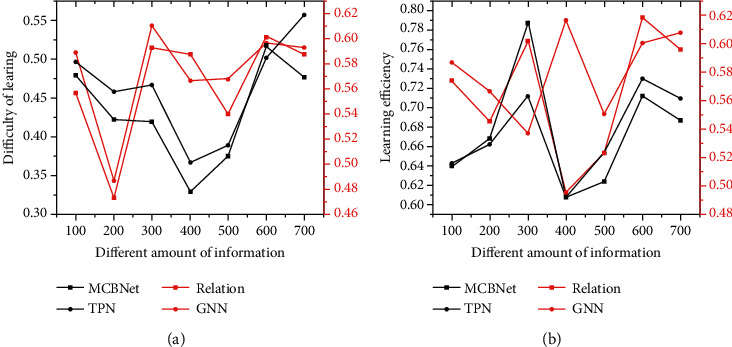
Comparison of learning capabilities of different models.

Figure 11 shows the comparison results of different learning methods. Figures 11(a) and 11(b) illustrate the comparisons of learning difficulty and learning efficiency, respectively. It is found that the learning difficulty and learning efficiency of the elderly show volatility changes as the amount of information increases. Compared with other models [33], the smart learning and memory model based on deep learning can synthesize a large number of target sample features, greatly decreasing the learning difficulty and improving the learning efficiency.

**Figure 12:**
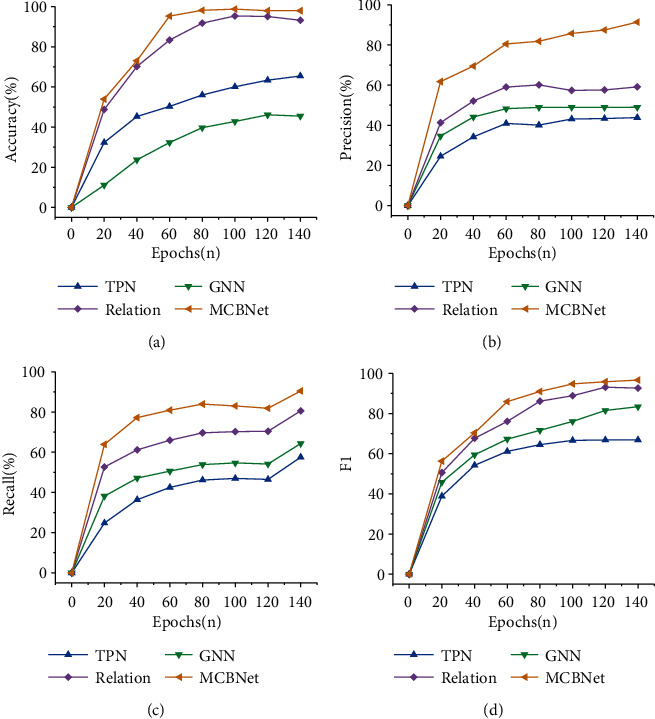
The accuracy rate, precision rate, recall rate, and F1 value changes in different algorithm models. (a) Accuracy rate; (b) precision rate; (c) recall rate; (d) *F*1 value.

The accuracy, precision, recall, and F1 value change curves of different algorithms are shown in Figure 12.

Figure 12(a) shows that the recognition accuracy of several algorithm models shows an overall upwards trend as the number of training periods increases. However, compared with other algorithms, the recognition accuracy of the proposed MCBNet algorithm increases faster. After 60 training periods, the accuracy rate reaches 98.75%, which is at least 3.15% higher than other algorithm models.  Figure 12(b) shows that among several algorithms, the accuracy of the MCBNet algorithm is consistently the best. When the accuracy rate of other algorithms decreases, the accuracy rate of the MCBNet algorithm maintains rapid growth.  Figure 12(c) shows that the recall rate of the MCBNet algorithm is at least 7.13% higher than that of the other algorithms.  Figure 12(d) shows that the *F*1 value of the MCBNet algorithm rises as the number of training periods increases. After the number of training periods is 100, the F1 value of the algorithm can reach 97.32%. The algorithm shows superior performance and can provide learners with higher accuracy rates for memory and recognition.

The research results show that many models, including the proposed image data classification solution, reach 99% accuracy. When the support sample of the category increases, the accuracy of all methods is lower than that of other tasks. When C64F-4 is adopted, the accuracy of the TPN solution improves by 1.4% and 2.4% on 5-channel 1-shot and 5-channel 5-shot, respectively. With the deepening of the feature extraction network, the accuracy of the low orbit increases by 0.4% and 1% on concurrent tasks, respectively. Compared with the previous models with better performance, it is of great significance to improve the accuracy of sample classification on the dataset.

## 5. Conclusion

Based on the learning characteristics and intelligent memory storage of the elderly, deep learning was adopted to design a smart device framework for the elderly, the learning efficiency and memory theory of the elderly were researched, and a home smart health management terminal suitable for the elderly was constructed. The comparative analysis of the experimental results on different image datasets and video datasets verified the effectiveness of the solution. This solution greatly reduces the difficulty of learning by integrating a large number of target sample features and further improves the memory and learning effects of the elderly. The main contribution of this study lies in the use of intelligent memory storage solutions under deep learning to study the design of smart devices for the elderly and provides corresponding research ideas for the production and design of smart devices in the IoT era. However, there are still some limitations in the article. First, the product is designed from the perspective of technical algorithms, and it needs continuous testing and optimization in the actual use process. Second, the designed product can effectively collect health data, but how to interconnect these smart devices in series to form a complete health analysis index system for the elderly is crucial to the establishment of the system. In the follow-up, in-depth research will be developed on these two aspects to continuously improve the effectiveness of the solution.

## Data Availability

The data used to support the findings of this study are available from the corresponding author upon request.
